# Urinary Stone Composition Analysis of 1465 Patients: The First Series from Azerbaijan

**DOI:** 10.34172/aim.32026

**Published:** 2024-11-01

**Authors:** Rashad Sholan, Rufat Aliyev, Ulduz Hashimova, Seymur Karimov, Elvin Bayramov

**Affiliations:** ^1^Scientific Research Center, State Security Service Military Hospital, Baku, Azerbaijan; ^2^A. Karayev’s Institute of Physiology, Azerbaijan National Academy of Sciences, Baku, Azerbaijan; ^3^Department of Kidney Diseases and Organ Transplantation, State Security Service Military Hospital, Baku, Azerbaijan; ^4^Department of Laboratory, State Security Service Military Hospital, Baku, Azerbaijan

**Keywords:** Azerbaijan, Calcium oxalate, Atone composition, Uric acid, Urinary stone disease

## Abstract

**Background:**

Urinary stone disease is a prevalent health issue worldwide, with varying incidence influenced by multiple factors. This study aims to provide the first comprehensive analysis of urinary stone composition in Azerbaijan.

**Methods:**

A retrospective study was conducted on 1465 patients, aged 1‒83 years, who underwent biochemical urinary stone analysis at the Department of Renal Diseases and Organ Transplantation, Azerbaijan State Security Service Military Hospital, between April 2015 and December 2023. Stone samples were analyzed using Fourier transform infrared (FTIR) spectroscopy. Statistical analyses were performed using the IBM® SPSS software version 29.0.

**Results:**

The cohort had a median age of 45 years, with a male-to-female ratio of 1.65:1. Calcium oxalate stones were the most common (56.2%), followed by uric acid (33.7%), struvite (5.3%), cystine (2.5%), calcium phosphate (1.9%), and xanthine (0.1%) stones. Men had a higher prevalence of calcium oxalate and uric acid stones, while women had more struvite stones. Mixed stones were common, particularly among uric acid and struvite stones. Significant differences in stone composition were observed between age groups and genders, with uric acid stones found predominantly in older individuals.

**Conclusion:**

This study highlights the predominance of calcium oxalate stones and the elevated prevalence of uric acid stones in Azerbaijan, emphasizing the need for tailored diagnostic and therapeutic approaches. The high prevalence of mixed stones underscores the complexity of urinary stone disease and the need for comprehensive metabolic evaluation and individualized preventive strategies.

## Introduction

 Urinary stone disease is a prevalent and significant health issue that adversely impacts human health.^1^ The global incidence of this disease ranges from 1% to 20%, and it is reported to be more common in men than women.^2-4^ Various factors are implicated in the formation of urinary stones, including gender, race, geographical location, occupation, hot climate, genetic predisposition, dietary habits (such as excessive consumption of caffeine, salt, dairy products, animal protein, and fat), smoking, alcohol consumption, physical inactivity, obesity, inadequate fluid intake, socioeconomic and educational status, water quality, excessive vitamin D intake, and metabolic disorders (such as diabetes mellitus, hypertension, and chronic kidney diseases).^5-7^ Additionally, studies suggest that low estrogen levels in postmenopausal women may increase the risk of kidney stone formation.^8^

 Although the precise mechanism of stone formation remains unclear, it is hypothesized that acidic urine pH contributes to the formation of calcium oxalate, uric acid, and cystine stones, whereas alkaline urine pH promotes the formation of calcium phosphate stones.^9^ The treatment approach is influenced by the size, location, degree of obstruction, and chemical composition of the stone.^10,11^ Urinary stones are categorized based on their composition as calcium oxalate, calcium phosphate, uric acid, struvite, cystine, and other types, with calcium stones accounting for approximately 70%‒80% of all stones.^4^ Factors such as metabolic syndrome, hypertension, body mass index, and kidney function have been associated with stone composition.^12,13^ Identifying the stone composition is crucial for both the treatment and prevention of the disease. Modifying dietary habits and ensuring adequate water intake are key strategies in preventing stone formation.^14^

 The Republic of Azerbaijan, situated in the South Caucasus region of Eurasia, encompasses both Western Asia and Eastern Europe. Despite the frequent occurrence of urinary stone diseases in clinical practice, scientific reports on this topic from our country are notably scarce. A national study from our neighboring country, Iran, has revealed a high incidence of urinary stones among Turkish/Azeri ethnic groups in the northwestern region of Iran.^15^ Interestingly, another Iranian study demonstrated that individuals of Turkish/Azeri ethnic origin have a greater susceptibility to recurrent episodes of urinary stones.^16^ In this study, we present the first comprehensive analysis of urinary stone composition from Baku, the capital of Azerbaijan.

## Patients and Methods

###  Patient Selection

 The study included 1465 patients, aged 1 to 83 years, who underwent biochemical urinary stone analysis at the Department of Renal Diseases and Organ Transplantation, Azerbaijan State Security Service Military Hospital, between April 2015 and December 2023. During the specified period, our hospital was the sole center in Azerbaijan capable of performing biochemical stone analysis. Of these patients, 96.2% (n = 1409) were referred to our center from external facilities for stone analysis, while 3.8% (n = 56) were diagnosed with urinary stones via ultrasonography or computed tomography after presenting to our hospital’s urology clinic. For patients with multiple results, only the first stone analysis was considered. Demographic data, including age, gender, and treatment modality, were retrieved from our hospital’s electronic database and sample admission forms. Treatment modalities were categorized as outpatient and inpatient. Indications for hospitalization included the development of stone-related pyelonephritis, severe renal colic, the need for IV hydration or antibiotics, and procedures such as percutaneous nephrolithotomy or extracorporeal shockwave lithotripsy for stones too large for medical management.

###  Stone Analysis

 Urinary stone samples were collected from various locations throughout Baku. Stones were retrieved after spontaneous passage (with or without medical therapy), surgery, or lithotripsy. Each sample was analyzed according to a standard operating procedure. The stone samples were ground and dried at 37 °C, then prepared into pellets with potassium bromide. Analysis was conducted using Fourier transform infrared (FTIR) spectroscopy (IR Affinity-1, Shimadzu Corporation, Japan). The composition of each stone was determined by comparing the FTIR spectra of the samples with a computerized library of reference spectra for single and mixed constituents. Each evaluation was conducted by qualified personnel and double-checked for accuracy.

###  Stone Classification

 Stones were classified following the guidelines of the European Urological Association and the Mayo Clinic stone classification practices.^14,17^ Mineral components accounting for at least 5% by weight were included in the analysis. Stones were classified as calcium oxalate if they contained more than 50% calcium oxalate, either in the form of calcium oxalate monohydrate or calcium oxalate dihydrate. Stones were categorized as calcium phosphate if they comprised over 50% apatite or brushite. Uric acid stones were identified if they contained more than 50% uric acid anhydrous, uric acid dihydrate, or ammonium urate. Stones with any presence of struvite were classified as struvite stones. Similarly, stones containing any amount of cystine were classified as cystine stones. Rare stone types (dolomite, whitlockite, aragonite) that did not fit into the specified categories, were classified as others.

###  Statistical Analyses

 Statistical analyses were conducted using the IBM® SPSS (Statistical Package for the Social Sciences) software version 29.0. Descriptive statistics were reported as frequency (percent) or median (minimum-maximum). The χ^2^ test was used to compare the proportions in different categorical groups. The Mann-Whitney U test was employed to compare two independent groups for non-parametric data. A 5% type I error rate was considered statistically significant.

## Results

**Figure 1:**
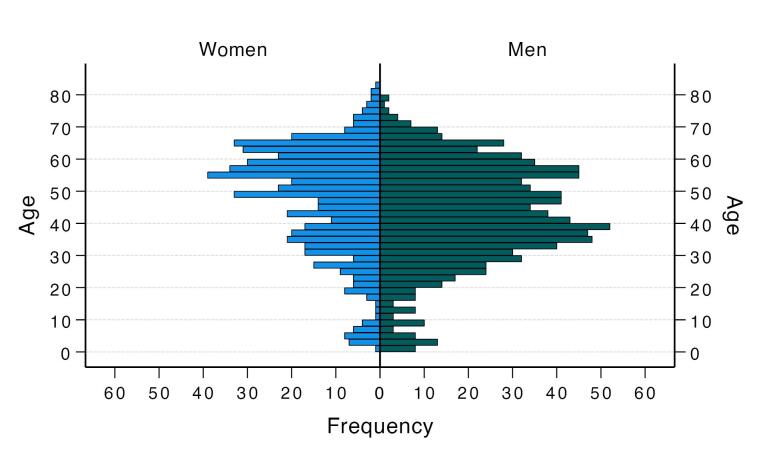
Age Distribution Pyramid of Men and Women Diagnosed With Urinary Stones

 A total of 1465 patients with a median age of 45 years (range: 1‒83) were included in the study. Among them, 913 (62.3%) were male, resulting in a male-to-female ratio of 1.65:1. The median age of women was significantly higher than that of men [51 years (range: 1‒83) vs. 42 years (range: 1‒79), *P* < 0.001, Figure 1]. Children and adolescents aged 1‒18 years constituted 7% of the patient population. Of the total cohort, 1409 (96.2%) were treated as outpatients, while 56 (3.8%) required inpatient care. Pure urinary stones, consisting of a single component, were found in 505 (34.5%) patients, whereas mixed stones, containing two or more components, were diagnosed in 960 (65.5%) patients. The distribution of urinary stone components across the entire cohort is detailed in Table 1.

**Table 1:** Distribution of urinary stone components (total 1465 patients)

**Stone Compositions**	**Frequency (Percent)**
Calcium oxalate	824 (56.2)
Calcium oxalate-monohydrate	784 (53.5)
Calcium oxalate-dihydrate	40 (2.7)
Uric acid	494 (33.7)
Uric acid anhydrous	462 (31.5)
Uric acid dihydrate	29 (2.0)
Ammonium urate	3 (0.2)
Struvite	78 (5.3)
Cystine	36 (2.5)
Calcium phosphate	28 (1.9)
Apatite	25 (1.7)
Brushite	3 (0.2)
Xanthine	2 (0.1)
Others	3 (0.2)

**Figure 2:**
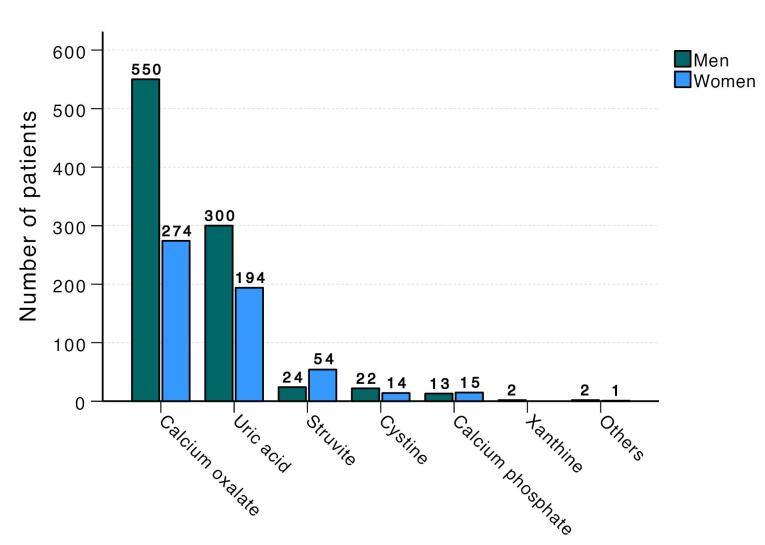
Frequency of Urinary Stone Components b Gender

 The most common stone type in men was calcium oxalate stones, accounting for 60.2%, followed by uric acid (32.9%), struvite (2.6%), cystine (2.4%), calcium phosphate (1.4%), and xanthine (0.2%) stones. In women, the most common stone type was also calcium oxalate, comprising 49.6%, followed by uric acid (35.1%), struvite (9.8%), calcium phosphate (2.7%), and cystine (2.5%) stones. The composition of urinary stones differed significantly between men and women (*P* < 0.001, Figure 2).

**Figure 3:**
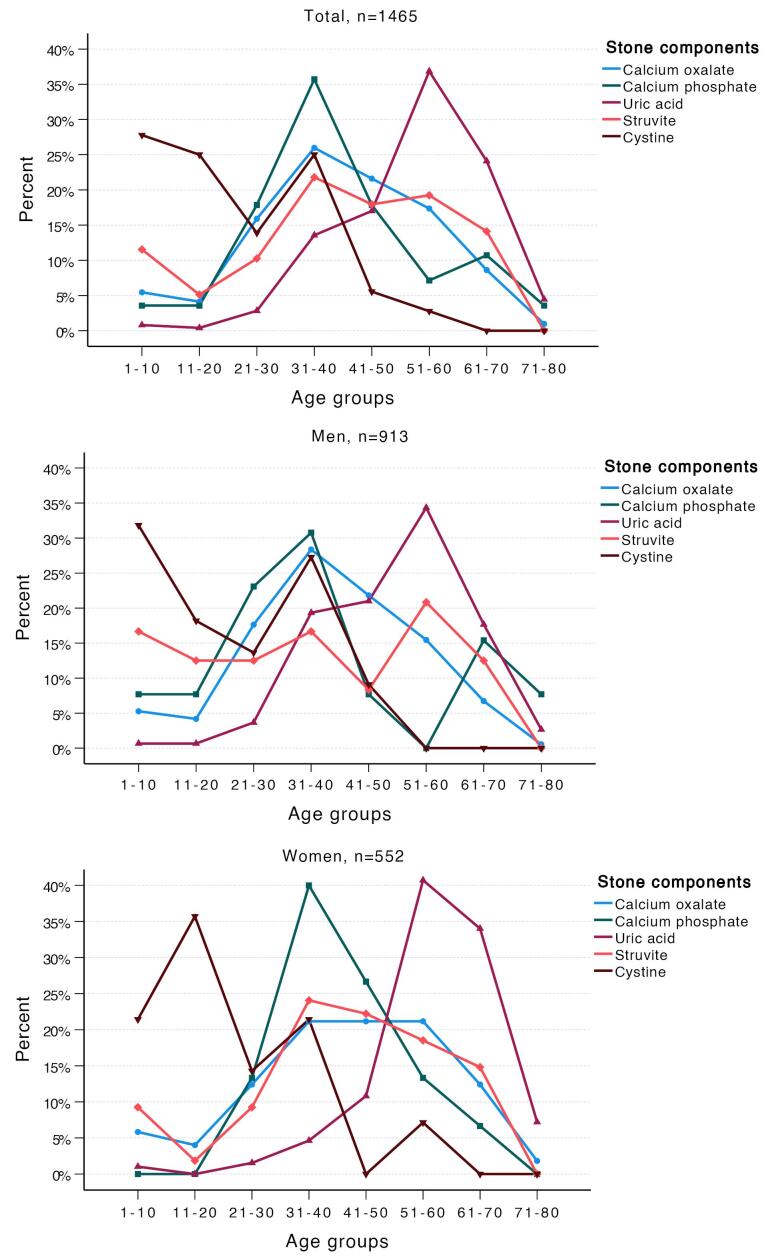
Percentage of Urinary Stone Components in the Entire Cohort (a), Men (b), and Women (c) Across Different Age Decades

 The distribution of urinary stones across different age groups is illustrated in Figure 3. Calcium oxalate and calcium phosphate stones peaked in the fourth decade of life, whereas uric acid stones were most commonly observed in the sixth decade. Struvite stones were predominantly found in individuals between the fourth and sixth decades of life, being most common in women during the fourth decade and in men during the sixth decade. Cystine stones were most frequently observed during the first two decades of life in both sexes. Following a second peak in the fourth decade, the frequency of cystine stones decreased with advancing age. The cohort was categorized into three age groups: under 18 years, 18 to 60 years, and over 60 years. The primary stone components differed significantly between these groups (*P* < 0.001, Table 2). While 29% of uric acid stones occurred in individuals over 60, 42% of cystine stones were found in patients under 18.

**Table 2:** Urinary Stone Components According to Patient Characteristics, Treatment Modalities, and Stone Types

**Characteristics, ** ***n*** ** (%)**	**Main Stone Components**					***P *** **Value**
	**Calcium Oxalate, ** ***n*** **=824**	**Uric Acid, ** ***n*** **=494**	**Struvite, ** ***n*** **=78**	**Cystine, ** ***n*** **=36**	**Calcium Phosphate, n=28**	
Gender						
Men	550 (66.7%)	300 (60.7%)	24 (30.8%)	22 (61.1%)	13 (46.4%)	< 0.001
Women	274 (33.3%)	194 (39.3%)	54 (69.2%)	14 (38.9%)	15 (53.6%)	
Age groups (y)						
< 18	61 (7.4)	6 (1.2)	11 (14.1)	15 (41.7)	1 (3.6)	< 0.001
18‒60	684 (83.0)	347 (70.2)	56 (71.8)	21 (58.3)	23 (82.1)	
> 60	79 (9.6)	141 (28.5)	11 (14.1)	0 (0.0)	4 (14.3)	
Treatment modality						
Outpatient care	791 (96.0%)	476 (96.4%)	75 (96.2%)	36 (100.0%)	26 (92.9%)	0.666
Inpatient care	33 (4.0%)	18 (3.6%)	3 (3.8%)	0 (0.0%)	2 (7.1%)	
Stone type						
Pure	389 (47.2%)	83 (16.8%)	17 (21.8%)	8 (22.2%)	5 (17.9%)	< 0.001
Mixed	435 (52.8%)	411 (83.2%)	61 (78.2%)	28 (77.8%)	23 (82.1%)	

 No statistical difference was found between stone components according to treatment modalities (outpatient vs. inpatient care, *P* = 0.666). Similarly, there were no significant differences in median age or gender across the treatment modalities (*P* = 0.765 and *P* = 0.757, respectively). More than half of all stone components were present in mixed stones. The proportion of mixed stones was 52.8% in calcium oxalate stones, 83.2% in uric acid stones, 78.2% in struvite stones, 77.8% in cystine stones, and 82.1% in calcium phosphate stones (*P* < 0.001). The frequency of pure or mixed stones did not differ according to gender (*P* = 0.409). The median age of patients with pure stones was 40 years (range: 2‒80), whereas the median age of patients with mixed stones was 47 years (range: 0‒83) (*P* < 0.001).

## Discussion

 This study provides a comprehensive analysis of urinary stone composition in a large cohort of patients from Baku, Azerbaijan, significantly contributing to the sparse literature on this topic from the region. The results highlight the predominance of calcium oxalate stones, which accounted for the majority of cases in both men and women, consistent with global trends.^18^ In a 2013 study involving,453 kidney stones from Turkey, calcium oxalate stones accounted for 80.4%, ranking first.^19^ In contrast, a recent study of 1092 samples from 10 cities in our southern neighbor, Iran, reported that 47.8% of urinary stones contained calcium.^20^ Similarly, a report from Israel last year found that calcium oxalate monohydrate stones were present in 51.9% of cases.^21^ In our cohort, the prevalence of calcium oxalate stones was determined to be 56.2%.

 The higher prevalence of calcium oxalate stones in males aligns with existing literature suggesting a gender disparity in stone formation, potentially linked to hormonal and metabolic differences.^22^ Androgens have been reported to increase oxalate excretion and promote calcium oxalate accumulation, while lower levels of estrogen in men facilitate oxalate formation in the urinary system. Conversely, women exhibit lower testosterone levels and higher urinary citrate levels, which are protective against stone formation.^2,23^ A study conducted by Roswitha Siener in 2022 reported a male-to-female ratio of 2.45:1 in a large German cohort.^24^ This rate was observed as 2.16:1 in the Turkish cohort^19^ and 2.06:1 in the Iranian cohort.^20^ Our cohort demonstrated a male-to-female ratio of 1.65:1.

 The median age of stone formation differed significantly between genders, with women presenting with stones at an older median age compared to men. This observation could be influenced by hormonal changes, particularly the reduction in estrogen levels post-menopause, which is known to impact stone risk.^22^

 Elevated oxalate levels in urine increase the saturation of calcium oxalate, leading to stone formation in conjunction with hypercalciuria.^22^ Adequate water consumption is suggested to prevent crystal formation in urine and subsequent stone formation.^25^ Conditions such as urine pH greater than 6.8, hyperparathyroidism, renal tubular acidosis, and urinary tract infections contribute to the formation of carbonate apatite (calcium phosphate carbonate) stones. The acidity of urine plays a role in the formation of uric acid stones, with factors such as gout, insulin resistance, lactic acidosis due to exercise, and high-protein animal foods contributing to their development.^26,27^ Urinary system infections, inflammatory bowel diseases, potassium deficiency due to the use of laxative drugs, and malnutrition are factors in the formation of ammonium urate stones. Magnesium ammonium phosphate (struvite) stones occur due to infections and are formed in the presence of bacteria that break down urea in the urine, and cystine stones are associated with cystinuria.^28^ In our study, the composition was found to be 56.2% calcium oxalate, 33.7% uric acid, 5.3% struvite, 2.5% cystine, 1.9% calcium phosphate, and 0.1% xanthine. The differences in proportions compared to other studies may be attributed to various factors such as study methods, regional climate, water composition, and nutritional conditions. Notably, the significantly higher frequency of uric acid stones in our cohort is striking, likely due to the increased prevalence of metabolic syndrome, abdominal obesity, and physical inactivity in our country in recent years.^29^

 Age-specific analysis revealed distinct patterns in stone composition. The peak incidence of calcium oxalate and calcium phosphate stones in the fourth decade of life, and uric acid stones in the sixth decade, aligns with previous studies indicating age-related metabolic changes affecting stone composition.^30,31^ Uric acid stones were predominantly observed in older individuals, particularly those aged 45 and above. Medical conditions such as diabetes, hypertension, ischemic heart disease, and obesity have been associated with the formation of these stones.^21^ Conversely, cystine stones were more prevalent in younger patients, especially in the first two decades of life, reflecting the genetic basis of cystinuria, a hereditary disorder leading to recurrent stone formation.^20,32^

 The significant variation in stone composition between genders is noteworthy. Men exhibited a higher frequency of uric acid stones, whereas women had a higher prevalence of struvite stones. Struvite stones, often associated with urinary tract infections, were more common in women, which is consistent with the higher incidence of urinary infections in females.^33^ On the other hand, benign prostatic hyperplasia (BPH) is a leading cause of lower urinary tract obstruction in older men, with bladder outlet obstruction resulting from BPH often contributing to urinary tract infections.^34^ In a study conducted in China by Wang et al, it was found that men had a higher prevalence of calcium oxalate stones and uric acid stones at the ages of 30‒49 compared to women. Conversely, women had a higher prevalence of infection stones and calcium phosphate stones at the ages of 30‒49 and 60‒69 compared to men. Additionally, the prevalence of uric acid stones increased with age in both sexes, while the prevalence of infection stones decreased.^35^ These gender-specific differences in stone composition necessitate tailored diagnostic and therapeutic approaches.

 The high prevalence of mixed stones, particularly among uric acid and struvite stones, underscores the complexity of urinary stone disease and the necessity for comprehensive metabolic evaluation in patients. The significant proportion of mixed stones suggests multiple underlying etiologies and reinforces the need for individualized dietary and pharmacological interventions aimed at addressing specific metabolic abnormalities.^14^ Preventive strategies should emphasize the importance of adequate hydration, dietary modifications, and management of metabolic conditions such as diabetes and hypertension, which are known risk factors for stone formation.^22^ Public health initiatives focusing on lifestyle modifications, including reducing the intake of high-risk dietary components and encouraging physical activity, could play a pivotal role in reducing the incidence of urinary stones in the population.

 The limitations of this study include its single-center design, which may limit the generalizability of the findings, and its retrospective nature, which could introduce biases related to data accuracy and completeness. Additionally, the lack of longitudinal follow-up data prevents assessment of recurrence rates and long-term treatment efficacy. The study’s demographic representation may not fully capture the diversity of the broader population in Azerbaijan, and it does not account for specific environmental or dietary factors influencing stone formation. The geographic focus on Baku may not reflect conditions in rural or other regions, and the absence of detailed metabolic evaluations limits understanding of underlying disorders.

 In conclusion, this study provides valuable insights into the epidemiology and composition of urinary stones in Azerbaijan, highlighting significant age and gender-related differences. The findings emphasize the importance of personalized management strategies based on stone composition and demographic factors. Further research is warranted to explore the underlying metabolic and genetic mechanisms contributing to stone formation and to develop targeted preventive and therapeutic interventions.
